# Zoonotic *Mansonella ozzardi* in Raccoons, Costa Rica, 2019–2022

**DOI:** 10.3201/eid3009.231415

**Published:** 2024-09

**Authors:** Joban Quesada, Paula Alfaro-Segura, Alberto Solano-Barquero, Karen Vega, Ernesto Rojas-Sánchez, Mauricio Jiménez, Alicia Rojas

**Affiliations:** University of Costa Rica, San José, Costa Rica (J. Quesada, P. Alfaro-Segura, A. Solano-Barquero, A. Rojas);; National University of Costa Rica Hospital for Minor and Wild Species, Heredia, Costa Rica (K. Vega, E. Rojas-Sánchez, M. Jiménez)

**Keywords:** *Mansonella ozzardi*, raccoons, Costa Rica, *Procyon lotor*, Central America, nematode, filarioid, zoonoses, parasites

## Abstract

*Mansonella ozzardi,* a filarioid parasite, causes human mansonellosis in the Americas. We identified raccoons (*Procyon lotor*) as wildlife reservoirs of *M. ozzardi* in Costa Rica. Noting the sympatry of free-ranging raccoons and humans, we conclude that mansonellosis is a considerable public health risk in the region.

*Mansonella ozzardi* is a nematode belonging to the family Onchocercidae and the etiologic agent of human mansonellosis in the Caribbean and Central and South America (*1*). *M. ozzardi* adult worms infect subcutaneous tissues of humans and release their microfilariae into the blood, which can be ingested by biting midges or ceratopogonids that, once infected, can then transmit the parasite (*2*). Human mansonellosis is usually asymptomatic, but researchers have reported fever, joint pain, headache, lower-limb chills, cutaneous rashes, and keratitis (*2*). Although humans are known to be the only natural, definitive hosts of the parasite, a study submitting patas monkeys (*Erythrocebus patas*) to experimental infection with *M. ozzardi* (*3*) suggested the possible presence of wild reservoirs.

Raccoons act as reservoirs for an assortment of zoonotic pathogens, including *Baylisascaris procyonis*. Procyonids have expanded their geographic range into urban areas because of forest fragmentation and loss of their sylvatic habitat (*4*), leading to increased contact with humans. The only *Mansonella* spp. reported in raccoons were *Mansonella llewellyni* parasites, detected in blood samples from the United States (*5*).

## The Study

We analyzed the internal transcribed spacer 1 (ITS1) and 12S loci of circulating microfilariae collected from blood samples of raccoons in Manuel Antonio National Park (Quepos, province of Puntarenas) and surrounding areas in Costa Rica. Raccoons were captured for routine health evaluation during 2019–2022. Wildlife specialists captured raccoons by using Tomahawk traps in areas where local persons and park rangers reported a high frequency of raccoons. Researchers then sedated the raccoons and collected blood samples. Sample collection, procedures, and analyses were approved under permits R-CM-UNA-005–2021-OT-CONAGEBIO and SINAC-PNI-ACOPAC-021–2019. Laboratory technicians stored the blood at 4°C prior to subsequent analysis. Raccoons were released once they fully recovered from the anesthesia.

We performed microscopic assessment of all blood samples by using the Knott modified technique (Appendix). We then extracted DNA from blood samples by using the DNeasy Blood and Tissue Kit (QIAGEN, https://www.qiagen.com), according to the manufacturer’s instructions. We then amplified mitochondrial 12S rRNA by using 12S.C345.F and 12S.C345R primers (*6*) and amplified the nuclear ITS1 loci with rDNA2 and rDNA158S primers (*7*). All reactions included *Toxocara canis* DNA as a positive control and a nontemplate control with PCR-grade water. We examined obtained amplicons on 1.5% agarose gels, purified positive reactions with Exo-SAP, and sequenced in both directions according to the Sanger method using the ABI 3730xl System (Macrogen Inc., https://dna.macrogen.com).

We used MEGA 7.0 software (https://www.megasoftware.net) to inspect and trim ITS1 sequences and aligned our sequences with other *Mansonella* spp. sequences available in GenBank by using the MUSCLE algorithm. We constructed a Bayesian inference tree by using the BEAST package (https://beast.community) and drew a Templeton Crandall-Sing haplotype network. Finally, we calculated Nei’s genetic distance for the ITS1 sequences (Appendix).

We observed microfilariae in 23.5% (4/17) of the raccoon blood samples in concentrations ranging 200–800 microfilariae/mL (Table). We noted the morphology of the observed microfilariae to be compatible with *M. ozzardi* original descriptions: 180–190 µm long and 3–4 µm wide; no sheath; tail elongated and slender without nuclei (Appendix) (*8*).

**Table:** Molecular and microscopic findings of *Mansonella ozzardi* in raccoons (*Procyon lotor*) from Manuel Antonio National Park and surroundings, Costa Rica, 2019–2022*

Sample identification	Sex of host	ITS1 loci results	12S rRNA gene results	Knott’s test results, microfilariae/mL
1P	F	Negative	Negative	200
2CP	F	LQS	97% ID, 95% QC†	600
MP11	M	99.02% ID, 100% QC‡	99.02% ID, 100% QC†	Negative
MP12	M	99.1% ID, 100% QC‡	LQS	Negative
MP14	M	97.02% ID, 100% QC‡	LQS	800
MP16	M	Negative	98.68% ID, 100% QC†	400
MP17	M	97.37% ID, 100% QC‡	97.52% ID, 100% QC†	Negative
MP18	M	98.27% ID, 100% QC‡	LQS	Negative

*ID, percentage of identity; ITS, internal transcribed tracer; LQS, low-quality sequence; QC, query coverage. †When compared with 12S sequence from Brazil (GenBank accession no. LT623914). ‡When compared with ITS1 sequence from Brazil (GenBank accession no. MN432519).

DNA could be isolated from 17 blood samples. In molecular analyses, 3 of 4 raccoons scoring positive at Knott’s test also demonstrated positive PCR tests, and 4 of 7 raccoons that tested negative at Knott’s test drew positive results by ITS-1 and 12S PCRs. Five of the 17 blood samples tested positive in the ITS1 PCR, and 4 of the 17 samples tested positive in the 12S PCR (Table). ITS1 sequences obtained from our analyses were 397–441-bp long and 97.0%–99.1% similar, with 100% of coverage with *M. ozzardi* isolated from humans in Brazil (GenBank accession no. MN432519). The 12S sequences were 107–151-bp long and 97.1%–99.1% similar to *M. ozzardi* from Brazil (GenBank accession no. LT623914.1). We deposited our new sequences into GenBank (accession nos. OR636492–6 for ITS1 and OR700019–22 for 12S).

*Mansonella* spp. clustered according to the nominal species in the ITS1 phylogenetic tree. Moreover, the *M. ozzardi* sequences we generated clustered in a separate clade in the BI tree, haplotype network and Nei’s genetic distance principal component analysis. The haplotype network revealed 6 *M. ozzardi* haplotypes: 1 from Costa Rica, 2 from Japan, 2 from Brazil, and 1 from Argentina (Figure).

**Figure:**
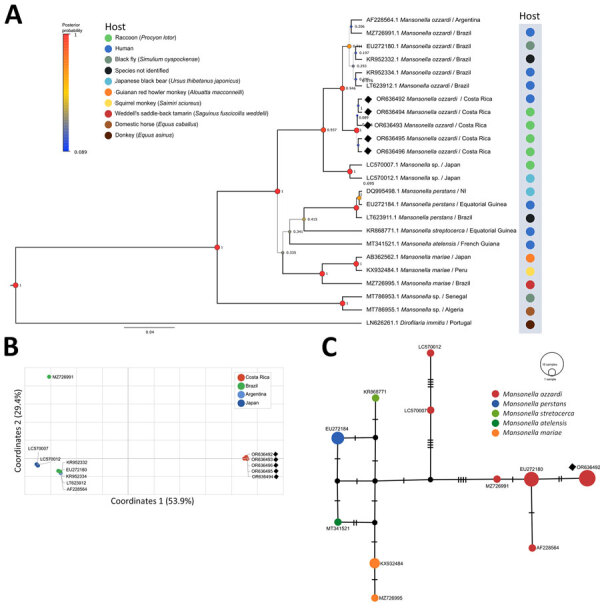
Phylogenetic and haplotypic analysis of *Mansonella ozzardi* ITS1 sequences obtained from racoons (*Procyon lotor*) in Costa Rica, 2019–2022 (black diamonds). A) Bayesian inference phylogenetic tree of *Mansonella* spp. based on the Hasegawa-Kishino-Yano with gamma distribution model. Line thickness and node size are proportional to posterior probability values. B) Principal component analysis of Nei’s genetic distance of *M. ozzardi* sequences from different geographic locations. C) Templeton Crandall Sing haplotype network of *Mansonella* spp. sequences. Black circles indicate hypothetical haplotypes; hatchmarks indicate mutational steps between haplotypes. GenBank accession numbers are shown for all sequences.

*M. ozzardi* is a filarioid nematode affecting humans in the Americas. Human mansonellosis is not completely harmless, and ocular impairment has been reported in humans from Panama (*9*). The lack of specific symptoms associated with mansonellosis usually leads to underdiagnosed infections and inadequate treatment (*10*). Accurate and prompt detection of the disease is therefore critical to avoid complications.

In our study, as in prior reports, PCR provided higher sensitivity compared with microscopic methods because molecular tools target DNA sequences rather than whole parasitic structures (*11*,*12*). Our results were as expected except for 1 sample determined to be positive by Knott’s test and negative in PCRs, possibly due to a prolonged period at room temperature that might have affected DNA quality. We attempted amplification of larger fragments of other genes but failed due to the presence of fragmented DNA.

Our findings suggest that raccoons are potential definitive hosts of *M. ozzardi*. Confirming the role of these procyonids in the life cycle of this worm*,* however, requires investigation into the anatomic location of adult worms inside the animals, the reservoir capacity of raccoons, and the feeding preference of intermediate hosts to raccoons. The *Simulium sanguineum* black fly, found in Panama, is the only simulid species reported thus far in Central America to act as intermediate host of *M. ozzardi*, but this simulid has not been reported in Costa Rica (*13*). Nevertheless, other *Simulium* spp. flies have been detected in this country, suggesting a need for more research to track infection status of such species and to determine the potential intermediate hosts used by *M. ozzardi* in Costa Rica.

Sympatry of raccoons and humans in densely populated regions has become a public health problem that can increase pathogen spillover to humans (*14*). Costa Rica is not the only country facing the expansion of raccoon populations into urban areas, largely due to human invasion of wildlife habitats resulting in ecosystem fragmentation. Several countries in Europe have reported a rise in the number and geographic range of racoons into human-inhabited environments. This expansion may increase pathogen spillover and the transmission of perilous diseases or parasites, such as *M. ozzardi*, to both domestic animals and humans (*15*). In Costa Rica, raccoons are reservoirs and carriers of such pathogens as *Salmonella* (*14*) and *B. procyonis*, one of the causing agents of visceral larva migrans (*4*). The encroachment of raccoons into urban areas thus represents a patent zoonotic risk (*4*,*14*).

## Conclusions

Because our findings implicate raccoons as potential reservoirs of the *M. ozzardi* parasite, and given the scarcity of information on intermediate hosts, we recommend active surveillance of *M. ozzardi* infections in humans. Our findings also support the need for histopathological studies to determine the location of adult *M. ozzardi* parasites in infected procyonids and further investigation into possible intermediate hosts and their feeding preferences to *P. lotor*. Such investigations will be crucial to providing a full understanding of the lifecycle of *M. ozzardi* in Costa Rica.

Appendix More information for zoonotic *Mansonella ozzardi* in raccoons, Costa Rica, 2019–2022.
