# Supplementary material for: Zoonotic Mansonella ozzardi in Raccoons, Costa Rica, 2019–2022
Source: Emerg Infect Dis. 2024 Sep;30(9):1930–3. doi: 10.3201/eid3009.231415 (PMC11346998; doi:10.3201/eid3009.231415)
Supplement: Appendix — More information for zoonotic Mansonella ozzardi in raccoons, Costa Rica, 2019–2022. [file 23-1415-Techapp-s1.pdf]

Article DOI: http://doi.org/10.3201/eid3009.231415

EID cannot ensure accessibility for supplementary materials supplied by authors. Readers who have difficulty accessing supplementary content should contact the authors for assistance.

Zoonotic *Mansonella ozzardi* in Raccoons, Costa Rica, 2019–2022

Appendix

Study Methods

Knott's modified technique

For this technique, 100 µL of blood were mixed with 900 µL of 2% formalin and centrifuged for 5 minutes at 1,500 rpm. The supernatant was discarded and 10 µL of the sediment was mixed with methylene blue in a slide to examine under a microscope at 100x and 400x magnifications. A sample was considered positive when microfilariae were observed. These were quantified and measured using the program ImageJ.

Bioinformatic analysis

Bayesian inference phylogenetic trees were built using the BEAST package (*1*) for ITS1 and 12S sequences. The best nucleotide substitution model was estimated with jModelTest (*2*) and chosen according to the Bayesian Inference Criteria (BIC). Accordingly, the Hasegawa-Kishino-Yano with gamma distribution was chosen as the best model for ITS1 sequences and the Tamura 3-parameter for the 12S sequences. Aligned sequences were uploaded into BEAUTi where 10^8 Markov chain Monte Carlo length and a burnin of 10% was set. Effective sample sizes of tree priors were verified with Tracer v1.7.2. Then, trees were summarized with TreeAnnotator v1.8 and visualized in FigTree v1.4.4.

Nei's genetic distance of ITS1 sequences was estimated and plotted into a Principal Component Analysis using GenAlEx v6.5 (*3*). In addition, a Templeton Crandall Sing (*4*) haplotype network was drawn using the PopArt software (*5*) using a 95% connection limit with *M. ozzardi, M. perstans, M. streptocerca, M. atelensis* and *M. mariae*.

References

1. Bouckaert R, Vaughan TG, Barido-Sottani J, Duchêne S, Fourment M, Gavryushkina A, et al. BEAST 2.5: An advanced software platform for Bayesian evolutionary analysis. PLOS Comput Biol. 2019;15:e1006650. PubMed https://doi.org/10.1371/journal.pcbi.1006650

2. Posada D. Selection of models of DNA evolution with jModelTest. Methods Mol Biol. 2009;537:93–112. PubMed https://doi.org/10.1007/978-1-59745-251-9_5

3. Peakall R, Smouse PE. GenAlEx 6.5: genetic analysis in Excel. Population genetic software for teaching and research—an update. Bioinformatics. 2012;28:2537–9. PubMed https://doi.org/10.1093/bioinformatics/bts460

4. Clement M, Posada D, Crandall KA. TCS: a computer program to estimate gene genealogies. Mol Ecol. 2000;9:1657–9. PubMed https://doi.org/10.1046/j.1365-294x.2000.01020.x

5. Leigh J, Bryant D. PopART: Full-feature software for haplotype network construction. Methods Ecol Evol. 2015;6:1110–6. https://doi.org/10.1111/2041-210X.12410

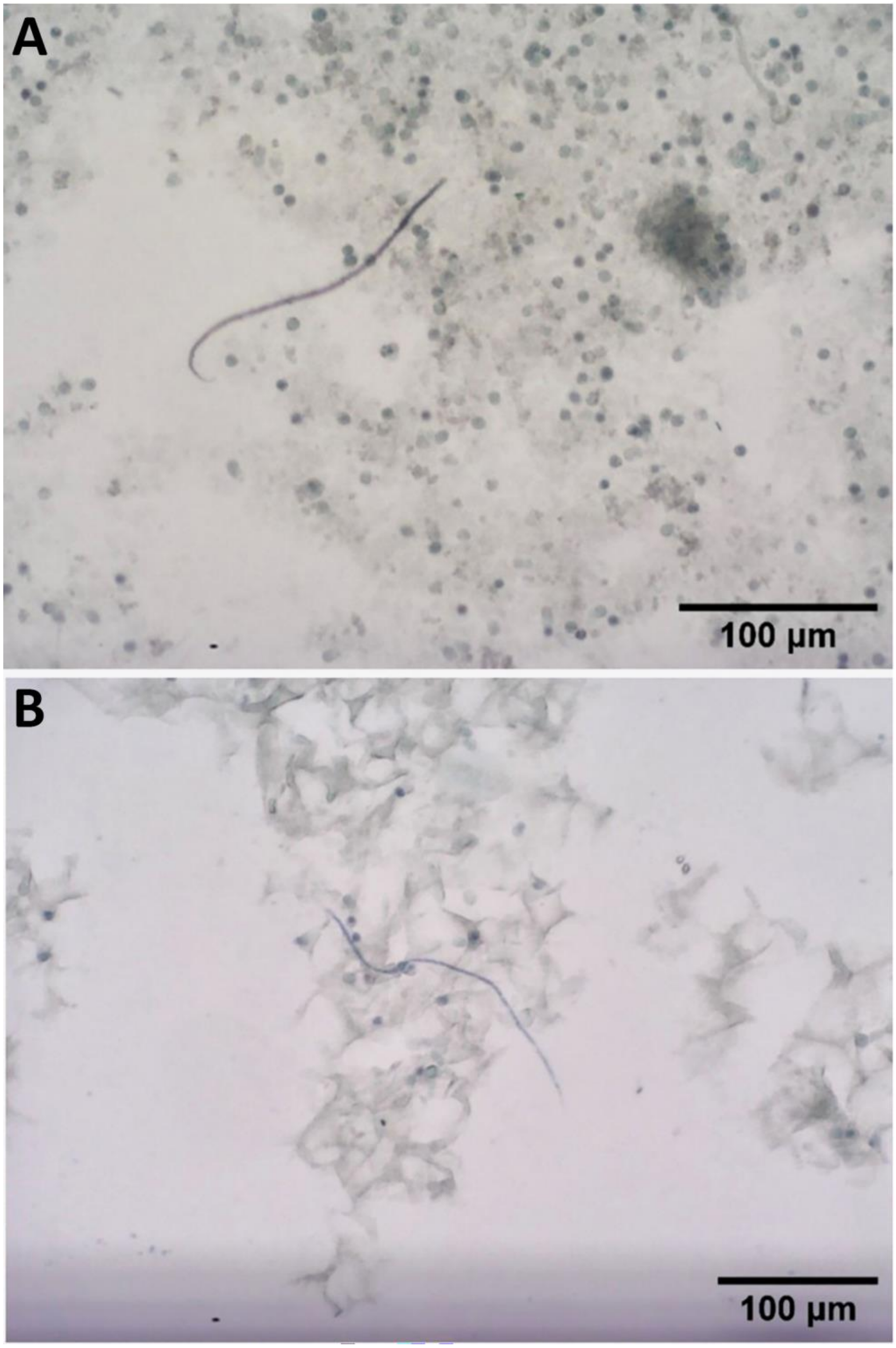

Appendix Figure. Laboratory results from a study of zoonotic *Mansonella ozzardi* in raccoons, 2019–2022, Costa Rica. A, B) Microfilariae obtained by Knott's method stained with methylene blue; original magnification x100.
